# A Prospective Study on Neural Biomarkers in Patients with Long-COVID Symptoms

**DOI:** 10.3390/jpm14030313

**Published:** 2024-03-16

**Authors:** Charikleia S. Vrettou, Alice G. Vassiliou, Chrysi Keskinidou, Panagiotis Mourelatos, Andreas Asimakos, Stavroula Spetsioti, Aristidis Diamantopoulos, Edison Jahaj, Archontoula Antonoglou, Paraskevi Katsaounou, Dimitra A. Vassiliadi, Anastasia Kotanidou, Ioanna Dimopoulou

**Affiliations:** 1 First Department of Critical Care Medicine, Evangelismos Hospital, Medical School, National & Kapodistrian University of Athens, 10676 Athens, Greece; alvass75@gmail.com (A.G.V.); chrysakes29@gmail.com (C.K.); silverakos@gmail.com (A.A.); edison.jahaj@gmail.com (E.J.); paraskevikatsaounou@gmail.com (P.K.); idimo@otenet.gr (I.D.); 2 Department of Endocrinology Diabetes and Metabolism, National Expertise Center for Rare Endocrine Diseases, Evangelismos Hospital, 10676 Athens, Greece

**Keywords:** long-COVID, s100b, GFAP, total Tau, health-related quality of life

## Abstract

Background: this prospective observational study aims to assess serum levels of glial fibrillary acidic protein (GFAP), s100b, and total Tau in long-COVID patients, exploring correlations with symptoms, cognitive decline, mental health, and quality of life. Methods: Long-COVID patients visiting our outpatient clinic (February 2021–December 2022) were screened alongside age- and sex-matched controls. GFAP, s100b, and total Tau in serum were measured with ELISA. Cognitive function, depression, anxiety, post-traumatic stress disorder, and quality of life were evaluated using MoCA, HADS (depression and anxiety), IES-R, and SF-36, respectively. Results: Sixty-five long-COVID patients and 20 controls were included. GFAP levels were significantly higher in long-COVID patients (*p* = 0.031), though not correlating with the presence of long-COVID symptoms. S100b and total Tau showed no significant differences between patients and controls. Nervous system-related symptoms were reported in 47% of patients. High rates of cognitive decline (65.9%), depression (32.2%), anxiety (47.5%), and post-traumatic stress disorder (44.1%) were observed. Over 80% of the study population scored below normative cutoffs for SF-36, indicating a significant impact on quality of life. Conclusions: in this long-COVID cohort with substantial psychological and cognitive symptoms, GFAP levels were elevated compared to controls, though not correlating with the presence of long-COVID symptoms.

## 1. Introduction

The COVID-19 global pandemic has undeniably exerted tremendous pressure on healthcare systems across the globe. With its primary impact on the respiratory system, SARS-CoV-2 can induce severe respiratory complications, posing significant challenges to medical facilities worldwide. However, alongside respiratory issues, the pandemic has also brought to light neurological symptoms associated with COVID-19, which were identified early in its emergence. Numerous researchers have meticulously documented various manifestations linked to the central nervous system (CNS) in COVID-19 patients. Additionally, heightened levels of neural injury biomarkers have been observed during the acute phase of the illness, indicating potential neurological involvement. Despite these findings, the precise pathophysiological mechanisms underlying CNS participation in COVID-19 remain shrouded in complexity and are not fully comprehended by the scientific community [1,2,3,4,5,6].

It is now widely recognized that the consequences of COVID-19 persist well beyond the acute phase of infection, giving rise to a spectrum of symptoms and signs collectively referred to as long-COVID syndrome. Beyond the initial illness, patients may experience an array of complaints, including but not limited to fatigue, dyspnea, chest pain, arthralgias, myalgias, dysautonomia, hair loss, or skin rash. Moreover, a constellation of symptoms related to the CNS may manifest, encompassing loss of taste and/or smell, mental fog, headache, sleep disturbances, depression, and anxiety. These symptoms can significantly diminish the quality of life experienced by affected individuals [7,8].

The pathophysiology of long-COVID syndrome is also incompletely understood. Proposed pathophysiological mechanisms include residual lung damage, direct invasion of CNS tissue, endothelial injury, and immune dysregulation [7,9]. Given that brain imaging typically does not reveal structural lesions in the majority of long-COVID cases, a more subtle CNS involvement is conceivable, potentially contributing to the elevation of neural injury biomarkers, as previously described in other neurological conditions, i.e., in the early stages of dementia and post-anoxic encephalopathies [10,11,12].

Several biomarkers of neural injury have been explored in the context of the long-COVID syndrome. Glial fibrillary acidic protein (GFAP), encoded by the GFAP gene in humans, is one such biomarker. It is expressed by diverse cell types within the CNS, including astrocytes and ependymal cells, especially during developmental stages. Despite being extensively studied as a cell marker, the exact function of GFAP remains largely unclear, though it is thought to play a role in maintaining astrocyte mechanical strength and cellular structure. The importance of GFAP extends across various critical CNS processes, including cell signaling, regulation of the blood–brain barrier, and CNS repair post-injury, notably in the formation of glial scars. With its potential as a blood biomarker for acute brain injuries across different disease mechanisms, GFAP has received considerable attention, also serving as a biomarker for secondary neural injury [13,14].

The protein s100b is a member of the intracellular, calcium-binding protein family primarily found in mature, perivascular astrocytes within the CNS, although it is also present to some degree in other CNS cells like oligodendrocytes and neural progenitor cells. Intracellularly, s100b plays a role in calcium homeostasis, facilitating signal transmission from second messengers. Additionally, it contributes to cell differentiation and cell cycle progression, with experimental evidence suggesting its ability to inhibit apoptosis. The diverse effects of s100b are believed to be mediated by the receptor for advanced glycation end-products (RAGE), whose upregulation by s100b may lead to the activation of proinflammatory genes [15]. However, much remains unknown about the precise mechanisms underlying s100b’s biochemical properties [15,16].

Outside the CNS, s100b can originate from various extracerebral sources such as Langerhans cells, adipocytes, epithelial cells, cardiac and skeletal muscle cells, and chondrocytes. Elevated serum s100b concentrations have been observed in patients with trauma involving multiple injuries to the thorax, extremities, and abdominal organs, even in the absence of identified CNS damage. S100b also holds clinical relevance in various cerebral conditions, including stroke, global ischemia, Alzheimer’s disease, and spontaneous subarachnoid hemorrhage, serving as a marker for injury severity. In contact sports, where mild and often repetitive traumatic brain injuries occur, s100b has emerged as a marker of brain injury severity, with its levels correlating with the ability to return to work [10,17]. However, it is worth noting that s100b levels can also be elevated in athletes without apparent head injuries, like swimmers and marathon runners, possibly due to extracranial release, posing a potential confounding factor. Given the short half-life of s100b, its elevation in the serum theoretically corresponds to a recent cerebral injury [16].

Tau proteins, derived from the gene microtubule-associated protein Tau through alternative splicing, constitute a set of six highly soluble isoforms known as tubulin associated unit (Tau) proteins. Their primary role involves maintaining microtubule stability within axons, with abundant presence in cerebral cortex neurons and minimal expression in CNS astrocytes and oligodendrocytes. When Tau proteins undergo hyperphosphorylation, they can form tangles of paired helical filaments and straight filaments, contributing to the development of tauopathies like Alzheimer’s disease and frontotemporal dementia [11]. Recent research has highlighted gender-specific expression patterns of the Tau gene across various brain regions, linking these differences to variations in tauopathy manifestations and risk factors. Moreover, individuals with Alzheimer’s disease or mild cognitive impairment exhibit higher levels of plasma total Tau (t-Tau) compared to control subjects. This elevated t-Tau in plasma holds promise as an early predictive biomarker for dementia, as prospective studies have shown associations between plasma t-Tau levels and dementia endophenotypes [11,18].

In an attempt to further investigate the role of CNS involvement in the symptoms of long-COVID, we designed a prospective observational study to assess serum levels of three well-described and previously used neural injury biomarkers measured in the serum, namely GFAP, s100b, and tTau, in individuals with long-COVID syndrome. Additionally, we aimed to investigate possible associations between CNS-related symptoms, cognitive performance, depression, anxiety, post-traumatic stress disorder (PTSD), and health-related quality of life (HQoL) scores with the levels of neural injury biomarkers.

## 2. Materials and Methods

### 2.1. Patients

The present prospective study enrolled patients who had tested positive for SARS-CoV-2 via polymerase chain reaction (PCR) and had undergone outpatient treatment or hospitalization, including admission to general wards or intensive care units (ICUs). These individuals sought evaluation at our long-COVID clinic between March 2021 and December 2022, at least 3 months after their initial COVID-19 diagnosis [7]. Exclusion criteria encompassed patients with a pre-existing history of neurological, neurosurgical, or psychiatric conditions. We documented all patients’ past medical history and all prescribed medications, including those prescribed before COVID-19 infection. A group of age and sex-matched healthy controls without a known history of COVID-19 infection was also included for biomarker measurements and comparisons with the long-COVID cohort. The study was approved by the Research Ethics Committee of the Evangelismos Hospital, Athens, Greece (No 112/31 March 2021); written informed consent was obtained from all patients before inclusion.

### 2.2. Blood Samples and Biomarker Measurements

Blood samples were obtained from all participants, and three biomarkers of neural injury were measured concurrently in the samples obtained from the patients presenting to the long-COVID clinic and from the healthy control group. Circulating levels of GFAP (intra-assay coefficient of variability (CV) 2.41%, detection limit 0.085 ng/mL (OriGene Technologies, Inc., Rockville, MD, USA), s100b (CV < 8%, detection limit 0.01875 ng/mL), and tTau (CV < 8%, detection limit 4.688 pg/mL) (Wuhan Fine Biotech Co., Ltd., Wuhan, China) were measured in serum samples by enzyme-linked immunosorbent assay (ELISA), according to the manufacturers’ instructions. The assays use two different polyclonal antibodies against the molecules as catching and tagging antibody. The researcher who performed the measurements was blinded to the samples measured.

### 2.3. Assessment of Cognition, Mental Health, and HQoL

Cognitive assessment was performed with the Montreal Cognitive Assessment (MoCA) scale. While different tests have been used for the assessment of cognition in studies involving long-COVID cases, the MoCA scale is the most frequently used tool [19]. The cutoff score is 26, with lower scores indicating cognitive impairment [20]. The following questionnaires were used for the assessment of mental health: (a) The Hospital Anxiety and Depression Scale (HADS) was used for the assessment of the severity of anxiety and depression. The HADS is a 14-question scale that measures depression and anxiety in the general medical population. The cutoff value is 7, with higher scores indicating significant symptoms of depression (HADS-d) or anxiety (HADS-a) [21]. (b) PTSD symptoms were assessed using the Impact of Events Scale–Revised (IES-R). Scores higher than 24 in the IES-R are considered to be of concern [22]. HQoL was evaluated using the 36-item Short Form Health Survey questionnaire (SF-36). The SF-36 is a widely used instrument for assessing HQoL, measuring eight scales that represent physical and mental dimensions: Physical Functioning (PF), Role Physical (RP), Bodily Pain (BP), General Health (GH), Vitality (VIT), Social Functioning (SF), Role Emotional (RE), and Mental Health (ME). The eight domains all contribute to the Physical Component Summary (PCS) and the Mental Component Summary (MCS) scores. A score of 50 is considered normative for all scales, with higher scores indicating better health status [23].

### 2.4. Statistical Methods

Data are presented as N (%), or median (IQR) for skewed variables. Correlations were measured with Spearman’s correlation coefficient (Sρ). Within-group comparisons were performed using the χ^2^ test for categorical variables or the Mann–Whitney test for ordinal variables. The sample size was estimated from previously published results. To observe a statistically significant difference (*p* < 0.05) in the tTau concentration between the long-COVID and the control group with 80% power, we would need 60 and 20 cases in the long-COVID and the control group, respectively [14]. The reduced number of healthy controls was predetermined due to the limited availability of age- and sex-matched adults without a documented history of prior SARS-CoV-2 infection at the time of enrollment, amidst the ongoing pandemic. Analyses were performed with the IBM SPSS statistical package, version 29.0 (IBM Software Group, New York, NY, USA), and with the GraphPad software, version 9.5.0 (GraphPad Software, LLC, Boston, MA, USA). *p* values < 0.05 were considered statistically significant.

## 3. Results

**Figure 1:**
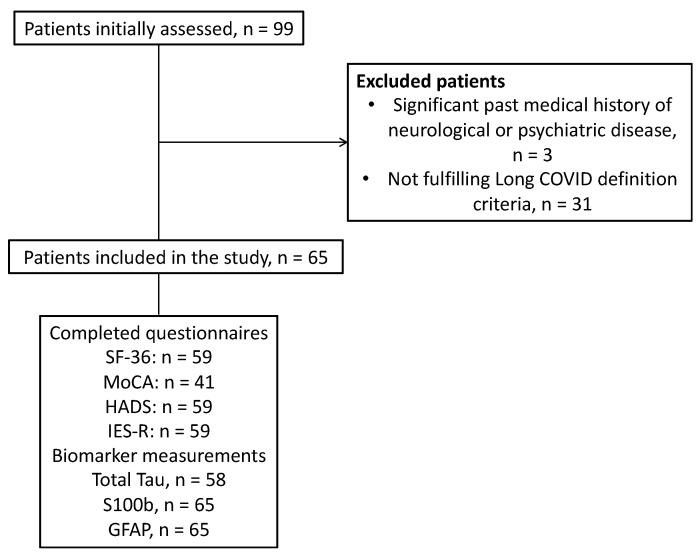
Study flowchart. COVID, coronavirus disease; GFAP, glial fibrillary acidic protein; SF-36, the 36-item Short Form Health Survey questionnaire; MoCA, Montreal Cognitive Assessment Scale; HADS, Hospital Anxiety And Depression Scale; IES-R, Impact Of Events Scale–Revised.

During the study period, 99 patients were examined in the long-COVID clinic of our hospital and 65 patients were eventually included in the study population. Neural injury biomarkers were also measured in twenty age- and sex-matched healthy controls. The study flowchart is shown in Figure 1. Table 1 presents the demographic and clinical characteristics of the study population. The majority (72.3%) of the long-COVID patients were hospitalized during the acute phase of illness, and 49.2% were also admitted in the ICU.

### 3.1. Long-COVID and Neural Biomarker Levels

**Figure 2:**
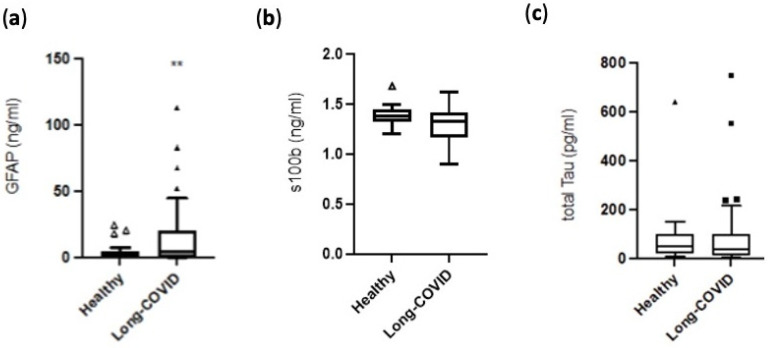
Levels of GFAP (**a**), s100b (**b**), and total Tau (**c**) in the long-COVID patients and in age and sex-matched normal controls. Biomarker levels are shown as box plots. Line in the box, median; box edges, interquartile range; whiskers, range of values; bullets, outliers. Comparisons were performed with the Mann–Whitney test. **, *p* < 0.05. GFAP, glial fibrillary acidic protein.

The levels of GFAP were significantly higher in the long-COVID patients than in the age and sex-matched controls [4.62 (0.22–20.23) ng/mL vs. 0.65 (0.08–5.20) ng/mL, *p* = 0.031]. There were no statistically significant differences in the serum levels of s100b [1.33 (1.17–1.41) ng/mL vs. 1.38 (1.32–1.45) ng/mL, *p* = 0.062] and tTau protein [38.08 (11.5–100.2) pg/mL vs. 45.0 (19.54–95.70) pg/mL, *p* = 0.631] between long-COVID patients and healthy controls. Figure 2 shows the distribution of serum biomarker levels in the long-COVID cases and the healthy controls.

### 3.2. Long-COVID Reported Symptoms

Fatigue was the most commonly reported symptom by the patients during the follow-up assessment (90.8%). Symptoms related to the nervous system were insomnia, confusion, loss of taste or smell, and headache, reported in 47% of patients (Table 1). There were no significant associations between biomarker levels and CNS-related symptoms (*p* > 0.05).

### 3.3. Long-COVID and Cognition, Mental Health, and HQoL

**Figure 3:**
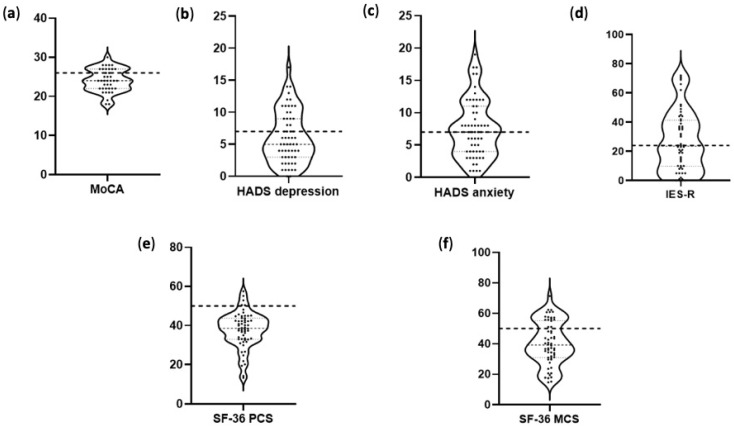
Violin charts showing the results of the tests used to assess cognition, mental health, and health-related quality of life. The horizontal dashed line refers to the cutoff of each questionnaire. The median (IQR) is shown within each plot. (**a**) MoCA test, (**b**) HADS-d scale, (**c**) HADS-a scale, (**d**) IES-R scale, (**e**) SF-36 PCS, and (**f**) SF-36 MCS. MoCA, Montreal Cognitive Assessment; HADS-d, Hospital Anxiety And Depression Scale—depression; HADS-a, Hospital Anxiety And Depression Scale—anxiety, IES-R, Impact of Events Scale—Revised; SF-36PCS, 36-Item Short-Form Health Survey questionnaire physical component summary; SF-36MCS, 36-Item Short-Form Health Survey questionnaire mental component summary.

Figure 3 shows the results of the administered questionnaires for cognition, depression, anxiety, PTSD, and HQoL. In the MoCA questionnaire, 65.9% of patients scored below the cutoff. Thirty-two percent (32.2%) of the population scored above the normal cutoff values in the HADS-d, and 47.5% in the HADS-a questionnaire. In the IES-R scale, 44.1% scored above the cutoff (Figure 3a–d).

The majority of the participating patients had significantly affected quality of life at three months after COVID-19 infection. The median (IQR) values for the eight components of the SF-36 in the long-COVID cohort were as follows: PF 36.65 (30.51–42.79), RP 35.15 (23.22–42.31), BP 40.47 (32.56–45.06), GH 43.52 (35.54–48.22), VIT 42.97 (33.99–51.95), SF 34.89 (29.51–45.65), RE (36.75 (25.39–48.11), and MH 38.50 (30.18–52.35). Figure 3e,f shows the violin charts of the SF-36 scores for the SF-36 PCS and the SF-36 MCS components. Notably, 89.8% of the study population scored below the cutoff in the SF-36 PCS and 74.6% scored below the cutoff in the SF-36 MCS.

There were statistically significant correlations between the SF-36 MCS and the HADS-d (S_ρ_ = −0.710, *p* < 0.001), the HADS-a (S_ρ_ = −0.685, *p* < 0.001), and the IES-R (S_ρ_ = −0.666, *p* < 0.001). Moreover, the HADS-d correlated with the HADS-a (S_ρ_ = 0.558, *p* < 0.001), and the IES-R (S_ρ_ = 0.627, *p* < 0.001). Finally, the IES-R also correlated with the HADS-a (S_ρ_ = 0.710, *p* < 0.001). There was a statistically significant, however weak, correlation between the levels of tTau and the MoCA score in the long-COVID cohort (S_ρ_ = 0.374, *p* = 0.028), and between s100b and the SF-36 PCS (S_ρ_ = 0.282, *p* = 0.031).

## 4. Discussion

The results of our study revealed heightened levels of GFAP among individuals suffering from long-COVID when compared to healthy controls who have no prior history of SARS-CoV-2-related illness. Conversely, there were no notable differences observed in the concentrations of s100b and tTau between these two groups. Despite GFAP being detectable in tissues outside the central nervous system, notably in the gastrointestinal tract, it remains firmly established as a prominent marker of CNS injury, with the brain serving as its primary origin. This underscores the significance of GFAP as a reliable indicator of CNS involvement, particularly in the context of long-COVID syndrome [13,14]. The elevated levels of GFAP detected in long-COVID patients provide further support for the hypothesis suggesting the intricate involvement of the CNS in the pathophysiology of long-COVID. These findings are consistent with previous research that has also observed heightened GFAP levels in long-COVID cohorts, without a concurrent increase in other neural injury biomarkers. This alignment reinforces the notion that GFAP may serve as a particularly sensitive indicator of CNS involvement in the context of long-COVID syndrome [24]. Significant associations between elevated GFAP levels and cognitive decline have been reported in female patients with long-COVID [25], whereas another study has documented lower GFAP levels in the blood of long-COVID individuals also experiencing persistent headaches [26]. Our findings, however, demonstrated that GFAP levels do not correlate with either the symptomatic manifestation of long-COVID or the performance of individuals in cognitive, mental health, and HQoL assessments.

Various clinical studies examining the role and progression of GFAP in long-COVID present conflicting findings, which could be attributed to variations in methodologies, including the severity of cases included, follow-up timeframes, and the specific long-COVID symptoms investigated. For instance, research by Needham et al. observed that sera from hospitalized COVID-19 patients showed elevated GFAP levels in a severity-dependent manner, suggesting ongoing brain injury even four months after follow-up [14]. However, in the study conducted by Telser et al., participants with moderate SARS-CoV-2 infections exhibited neurological symptoms that did not correlate with GFAP levels [24]. Additionally, De Boni et al. reported lower GFAP levels in blood samples from patients experiencing persistent headaches, suggesting that long-COVID-19 headaches might not indicate underlying neuronal damage or neuroinflammation [26]. Furthermore, Bark et al. explored the potential connections between serum GFAP levels, cognitive impairment, and fatigue in long-COVID patients. Cognitive function was evaluated using the MoCA scale, while fatigue was assessed using the Multidimensional Fatigue Inventory (MFI-20) [25]. Elevated GFAP levels occurring 3 to 6 months post-ICU discharge were linked to cognitive dysfunction, particularly among female patients. Conversely, patients experiencing fatigue at follow-up exhibited significantly lower GFAP levels. These findings suggest a potential association between GFAP levels and neuropsychiatric outcomes following acute COVID-19 that necessitated ICU care.

S100b has long been acknowledged as a sensitive biomarker for assessing brain damage and blood–brain barrier integrity, currently employed for risk stratification in mild brain injury [16]. In comparison to other biomarkers of neural damage, s100b has the disadvantage that it can be synthesized outside the CNS in acute trauma and shock, diminishing its specificity [27]. Despite its lack of specificity, s100b was discovered to be elevated in individuals with acute COVID-19 compared to controls, and it exhibited significant predictive capability for mortality in critically ill COVID-19 patients [28]. Furthermore, s100b concentration displayed significant correlations with inflammation markers (such as ferritin, C-reactive protein, and procalcitonin) as well as indicators of organ damage (such as alanine aminotransferase and creatinine) in acute COVID-19 populations [17]. Notably, s100b outperformed other biomarkers of neural injury, including neuron-specific enolase (NSE), particularly as a predictor of severity and mortality, as indicated by our prior research [28]. A sustained elevation of s100b in post-acute COVID-19 could theoretically be attributed to ongoing neuroinflammation, neuronal and astrocytic injury following COVID-19, or compromised brain oxygenation due to persistent lung disease. However, our current findings do not support either of these hypotheses [17,28].

In our study, no significant differences in s100b were observed between long-COVID cases and controls; s100b, however, exhibited weak, yet statistically significant, positive correlation with the SF-36PCS scores. Elevated levels of s100b have been observed in response to stress and physical exercise [10]. The factors that have been implicated in s100b increases include muscle breakdown, the level of training, and oxidative stress [29]. This latter association may explain the observed positive correlation between s100b and SF-36PCS within our cohort, as it is conceivable that individuals engaged in higher levels of physical activity may also experience more physiological stress, particularly during the convalescent phase following the disease.

Our study showed no differences in tTau levels between long-COVID patients and healthy controls. The absence of significant difference in the levels between long-COVID patients and normal controls has also been documented in previous studies [30]. Other researchers reported a distinctive pattern of evolution in tTau levels in long-COVID, differing from other biomarkers of CNS injury. Specifically, tTau levels were observed to increase during convalescence compared to levels in the acute phase [14]. This may have partially contributed to the weak, yet statistically significant positive correlation of tTau with the MoCA score that was observed in our cohort.

Lennol et al. found that individuals diagnosed with COVID-19 during the initial phase, yet lacking significant neurological manifestations, exhibited notably elevated levels of plasma tTau in comparison to control groups. However, these elevated plasma tTau levels returned to normal following recovery. Furthermore, when subdividing COVID-19 patients based on the presence of symptoms during re-evaluation three months post-acute phase, no difference in tTau levels was observed between patients and controls. In the same study, and in contrast to our cohort, the majority of included patients had only mild or moderate disease that did not require hospitalization [30].

In another study by Needham et al., an increase in serum tTau levels was observed in patients during follow-up, which seemed unrelated to the severity of the initial COVID-19 illness. Interestingly, this rise in serum tTau concentration was not only independent of disease severity but, upon further analysis correcting for multiple comparisons, it was found that only patients with mild COVID-19 disease had significantly higher tTau concentrations compared to controls, at follow-up. Additionally, an unexpected connection between serum tTau levels and HQoL assessments emerged, indicating that higher concentrations were associated with better scores, particularly in the emotional, well-being, and energy/vitality aspects of the SF-36 scale. However, it is important to note that none of these correlations remained significant after adjusting for multiple comparisons [14].

Since the levels of tTau have consistently been linked to cognitive decline in individuals diagnosed with dementia and serve as a diagnostic biomarker for Alzheimer’s disease [11], the unforeseen correlation in our cohort prompts the conclusion that cognitive dysfunction in long-COVID follows a distinct pathophysiology compared to dementias. There is accumulating evidence supporting the idea that cognitive impairments linked to COVID-19 infection persist even during the recovery period. These observed cognitive deficits varied across studies, but were not solely attributed to variations in respiratory symptom severity, nor were they explained by demographic or socioeconomic differences such as age or education. Notably, the most significant impairments were evident in cognitive tasks involving reasoning, problem-solving, spatial planning, and target detection, while simpler functions like working memory span and emotional processing seemed unaffected [31].

In addition to cognitive decline, our findings indicate that long-COVID exerts a substantial impact on mental health. The rates of abnormal scores in depression, anxiety, and post-traumatic stress assessment tools fall within the range reported in studies involving patients with acute respiratory distress syndrome (ARDS) of various etiologies and those diagnosed with post-intensive care syndrome (PICS) [8,32]. The effect on mental health is further mirrored in the prevalence of abnormal results in HQoL assessments. Given the absence of an association between mental health assessment results and the examined CNS injury biomarkers, we hypothesize that the effects of long-COVID may also stem from residual respiratory disease [33]. Previous research conducted on hospitalized patients with respiratory illness not only revealed measurable cognitive impairments, both objectively and subjectively, but also indicated that these deficits persist for some individuals even at a 5-year follow-up [34].

Overall mental health, and psychological stress, especially, are believed to harm cognition [35]. When stressors trigger the hypothalamic-pituitary-adrenal (HPA) axis, cortisol is released, binding to receptors in the hippocampus and prefrontal cortex. Prolonged and elevated cortisol levels have been linked to neurotoxic effects on these brain regions, contributing to cognitive impairments. Recent meta-analyses suggest that memory might be particularly vulnerable to high levels of neuroticism and perceived stress, but there is a research gap in areas like executive function that warrants attention in future studies [36]. An alternative explanation is that immune dysregulation associated with long-COVID contributes to the pathophysiology of long-COVID symptoms—a theory that has also garnered support in the context of ME/CFS, a clinical syndrome sharing numerous clinical similarities with long-COVID, including fatigue as the most prominent characteristic symptom [37]. Similar to long-COVID, ME/CFS is linked to previous viral infections and often manifests in individuals who were previously healthy and active. Both conditions exhibit a chronic pattern characterized by fatigue, cognitive impairment, and post-exertional malaise, significantly impacting daily activities. The absence of a definitive diagnostic test poses a significant challenge, often leading to diagnoses by exclusion or delayed recognition as ME/CFS, while some patients never receive a clear diagnosis. Current management options for ME/CFS are limited, with treatments for associated psychiatric conditions offering only marginal benefits [38]. Many patients with ME/CFS never fully regain their health, placing a substantial burden on their families who often assume lifelong caregiving responsibilities [38,39]. Given these, it is reasonable to consider that patients might benefit from early intervention with rehabilitation, physiotherapy, and psychological support in the early post-COVID-19 phase, even before fulfilling the three-month criterion for long-COVID diagnosis [7].

In our study, we conducted a comprehensive assessment of the impact of long-COVID on cognition, mental health, and HQoL. We measured three well-established CNS injury biomarkers—namely, GFAP, s100b, and tTau. However, it is important to acknowledge that our study has several limitations, the most prominent being the relatively small sample size. This limitation could have potentially hindered our ability to detect significant differences in biomarker levels between long-COVID patients and the control group. Furthermore, the lack of consecutive measurements for these biomarkers limits our ability to glean insights into the dynamic nature of CNS involvement in long-COVID over time. Another limitation arises from the inclusion of patients from a follow-up long-COVID clinic, which may introduce a selection bias. It is plausible that patients with more severe symptoms, who were unable to attend the clinic, may not be adequately represented in our study cohort. These limitations underscore the need for larger-scale studies with longitudinal assessments to further elucidate the complex interplay between long-COVID and CNS function.

To summarize, our study findings indicate a rise in GFAP levels among individuals with long-COVID, while no corresponding increase is observed in s100b and tTau biomarkers. However, despite this elevation in GFAP, we found no correlation between these biomarker levels and CNS-related symptoms, cognitive function, mental health, or health-related quality of life. While the pathophysiology of long-COVID does involve the central nervous system, it appears distinct from other neurological conditions, and the cognitive impairment associated with it does not align with dementia syndromes. Nevertheless, the significant impact of long-COVID on quality of life emphasizes the critical need for timely implementation of rehabilitative and psychological interventions to address the multifaceted challenges faced by affected individuals.

## Figures and Tables

**Table 1:** Demographics, comorbidities, and symptoms in long-COVID patients.

Characteristics	Value
Number of patients	65
Age (years)	56 (45–63)
Sex	
Male	36 (55.4%)
Female	29 (44.6%)
Hospitalization status during acute illness	
ICU	32 (49.2%)
Mechanical ventilation	29 (44.6%)
High-flow oxygen therapy	3 (4.6%)
Ward	15 (23.1%)
Outpatients	18 (27.7%)
Smoking habit at the time of enrollment	
Yes	8 (12.3%)
No	57 (87.7%)
Comorbidities	
Hyperlipidemia	22 (33.8%)
Hypertension	17 (26.2%)
Diabetes	8 (12.3%)
COPD	7(10.8%)
Cancer	5 (7.7%)
Coronary artery disease	3 (4.6%)
Chronic renal failure	1 (1.5%)
Long-COVID symptoms	
Fatigue	59 (90.8%)
Dyspnea	45 (69.2%)
Arthralgia	32 (49.2%)
Alopecia	30 (46.1%)
Insomnia	29 (44.6%)
Chest pain	20 (30.8%)
Cough	19 (29.2%)
Loss of taste/smell	19 (29.2%)
Headache	17 (26.1%)
Confusion	15 (23.1%)
Nausea	9 (13.8%)
Other symptoms *	9 (14.8%)

Note: Numeric variables are shown as median (interquartile range); categorical variables displayed as count (percent of total). * Other symptoms included abdominal pain, diarrhea, itching, and palpitations. COVID-19, coronavirus disease 2019; ICU, intensive care unit; COPD, chronic obstructive pulmonary disease; GFAP, glial fibrillary acidic protein.

## Data Availability

The data that support the findings of this study are available from the corresponding author upon reasonable request.
